# A machine learning approach for the identification of key markers involved in brain development from single-cell transcriptomic data

**DOI:** 10.1186/s12864-016-3317-7

**Published:** 2016-12-22

**Authors:** Yongli Hu, Takeshi Hase, Hui Peng Li, Shyam Prabhakar, Hiroaki Kitano, See Kiong Ng, Samik Ghosh, Lawrence Jin Kiat Wee

**Affiliations:** 1 Institute for Infocomm Research, A*STAR, 1 Fusionopolis Way, #21-01 Connexis (South Tower), Singapore, Singapore; 2 The Systems Biology Institute, Falcon Building 5 F, 5-6-9 Shirokanedai, Minato, Tokyo, 108-0071 Japan; 3 Computational and Systems Biology, Genome Institute of Singapore, A*STAR, 60 Biopolis Street, Genome, #02-01, Singapore, 138672 Singapore; 4 The Systems Biology Institute, Singapore Node hosted at the Institute for Infocomm Research, A*STAR, Singapore, Singapore

**Keywords:** Single-cell RNA-seq, Machine learning, Network reconstruction, Systems biology

## Abstract

**Background:**

The ability to sequence the transcriptomes of single cells using single-cell RNA-seq sequencing technologies presents a shift in the scientific paradigm where scientists, now, are able to concurrently investigate the complex biology of a heterogeneous population of cells, one at a time. However, till date, there has not been a suitable computational methodology for the analysis of such intricate deluge of data, in particular techniques which will aid the identification of the unique transcriptomic profiles difference between the different cellular subtypes. In this paper, we describe the novel methodology for the analysis of single-cell RNA-seq data, obtained from neocortical cells and neural progenitor cells, using machine learning algorithms (Support Vector machine (SVM) and Random Forest (RF)).

**Results:**

Thirty-eight key transcripts were identified, using the SVM-based recursive feature elimination (SVM-RFE) method of feature selection, to best differentiate developing neocortical cells from neural progenitor cells in the SVM and RF classifiers built. Also, these genes possessed a higher discriminative power (enhanced prediction accuracy) as compared commonly used statistical techniques or geneset-based approaches. Further downstream network reconstruction analysis was carried out to unravel hidden general regulatory networks where novel interactions could be further validated in web-lab experimentation and be useful candidates to be targeted for the treatment of neuronal developmental diseases.

**Conclusion:**

This novel approach reported for is able to identify transcripts, with reported neuronal involvement, which optimally differentiate neocortical cells and neural progenitor cells. It is believed to be extensible and applicable to other single-cell RNA-seq expression profiles like that of the study of the cancer progression and treatment within a highly heterogeneous tumour.

## Background

The advent of sequencing technology has brought about the unprecedented ability to sequence individual single cells. Now, the distinct gene expression profiles of seemingly similar yet genetically heterogeneous subpopulations of cells within different tissue types can be elucidated with the use of single-cell sequencing technology. The study of such subpopulations within tumours is especially important in the study of differential reactivity of patients to drug treatments and that of acquired drug resistance within cancer patients [1, 2]. The complex underlying transcriptomic dynamics elucidated will enhance our understanding of the distinct gene expression signatures of different carcinomas or subpopulations within disparate tumour tissues which will ultimately aid in the optimization of cancer treatments.

A major challenge, however remains, is that of a suitable computational analytic pipeline for the analysis of single-cell RNA-Seq transcriptomic data. To address this problem, this paper proposes the identification of the unique gene expression profile within each subpopulation through traditional statistical methodology, geneset enrichment analysis (GSEA), machine learning algorithms where genes identified are subsequently used to build predictive classifiers for cell type prediction. Computational analysis of RNA-Seq transcriptomic data using machine learning algorithms, particularly that of supervised learning algorithms, like rule-based machine learning techniques [3], Support Vector Machine (SVM)-based [4, 5] and network-based approaches [6], is not new. However, this paper is the first to utilize a combination of two different machine learning algorithms (SVM and Random Forest (RF)) on single-cell RNA-seq transcriptomic data to identify the key signatures of different cell types for cell type prediction. Using single-cell RNA-seq expression data from neocortical cells and those of neural progenitor cells as inputs, we have identified a set of 38 key genes which optimally differentiates developing neocortical cells and those of neural progenitor cells.

Further, relevance of the differentially expressed genes in neuronal cell differentiation were also investigated using network-based approaches where the gene regulatory networks (GRNs) inferred elucidated the potential underlying interactions/functions of the key hub genes (eg, genes that regulate many genes in neuronal cells but do not regulate genes in neuronal progenitor cells) which could be further validated in wet-lab experimentation [7, 8]. In summary, this paper described a novel computational pipeline for the study of single-cell RNA-Seq transcriptomic data where key genes identified were used, with high accuracy, to predict distinct neuronal cell subtypes where such a system could be used to uncover the different subpopulations within a newly sequenced brain tissue. In addition, downstream network studies lend a systems-level relevance where potential underlying relationships are unravelled and potentially be used for targeted for treatment in neuronal developmental diseases.

## Results

### Prefiltering of genes

**Fig. 1:**
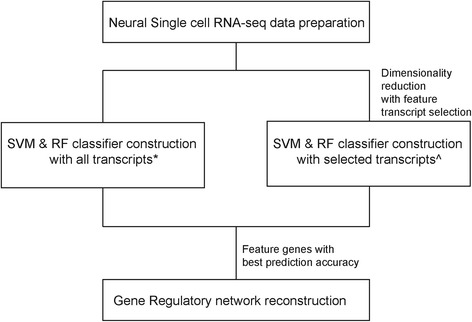
Workflow for data analysis carried out in this paper. *All genes refers to the set of genes filtered by expression values and ^ selected genes refers to the optimal set of genes identified by geneset enrichment analysis (GSEA), statistical and machine-learning approaches (See Methods for more information)

The summary of the methodology employed in this paper is summarized in Fig. 1.

**Fig. 2:**
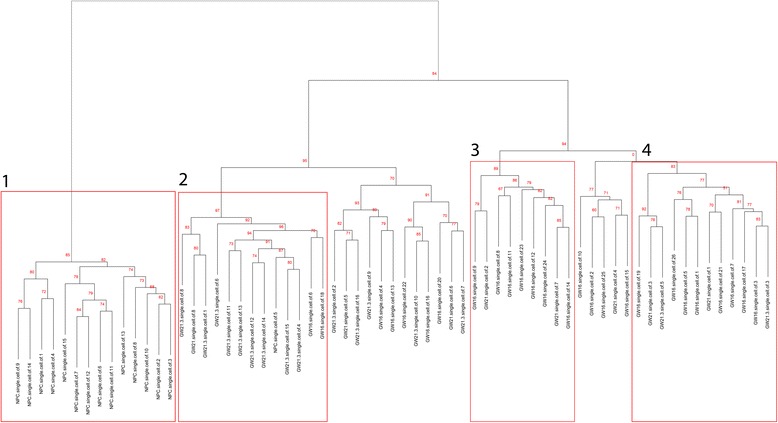
Clustering of 65 neuronal cells. The approximately unbiased (AU) probability value at each node is shown in red font. There are four distinct clusters (red boxes labelled 1–4) with an AU higher than 80. Box1 comprises of mainly NPCs while boxes 2–4 primarily consists of neuronal cells only

Raw data, downloaded from the NCBI databased, was filtered (the criteria used for data filtering is reported in the materials and methods section) to obtain a total 8681 (~15%) genes which represents the dataset denoted by “all genes”. A total of 65 samples (15 NPC and 50 developing neuronal cells) were used as training data to SVM and RF algorithms. Significance of the cell types assignment is further validated using Pvclust [9], which employs a multiple bootstrap (50,000) resampling algorithm to calculate the approximately unbiased (AU) probability values for cluster distinctions which is shown in red in Fig. 2.

### SVM and RF-based classification of neuronal cell types on all expressed genes

Each set of gene expression data, extracted from different feature selection methods, was used to train two different machine learning (ML) models, namely a SVM and a RF classifier. The classification was reduced to a two-class problem where the predictor was designed to identify potential neocortical neuronal cells from NPCs.

Due to the limited number of samples, 65 (15 NPC and 50 neuronal cells) used in this study, the data was not separated into training and testing set for the construction of SVM-based classifiers. Instead, leave-one-out (LOO) cross validation was carried out. However, this was deemed unnecessary for RF as classifiers were built by aggregating a large number of different decision trees, predictors built with the random forests algorithm is expected to have low variance and low bias.

SVM and RF classifiers built with the filtered high dimensional single-cell RNA-seq expression dataset, consisting of more than 8000 transcripts (Table 1), yields an accuracy of 95.3 and 76.9% respectively (Table 2). It seems like classifiers built with all transcripts, sans those of low expression, are able produce classifiers of a reasonably high accuracy, however, the quality of such classifiers needs to be co-ordinately investigated. To this end, the Matthews correlation coefficient (MCC) was used to validate the quality of the classifiers constructed. A coefficient of 1 represents a prefect prediction while that of 0 indicates a classifier producing predictions similar to random prediction. The SVM classifiers were far superior to the cognate RF classifiers having a MCC of 0.91 and 0, respectively. Thus, there is a need for additional feature transcript selection process to enhance both the accuracy and the quality of the constructed classifiers. Additionally, the construction of classifiers based on all transcripts are computational inefficient and the inclusion of large number of “noisy genes” will obscure important underlying signatures of each phenotypic class due to data overfitting and this will greatly limit the accuracy and quality of the classifiers [10]. On a more biological note, such a method fails to identify a subset of key genes which might have important biological applications in novel biomarker discovery.

**Table 1:** Genes/features selected by disparate feature selection techniques

Feature selection techniques^a^	Features/Genes (No.)
Filtered by low expression	8281
GSVA feature enrichment	1161
sRAP	837
SVM-RFE	38
RF-based Positive MDA	3339
*T*-test	60

^a^Feature selection is based on five different methodologies based on machine learning algorithms (SVM and RF) and also that of traditional differentially expressed genes (*sRAP*), *t*-test based analysis (*limma*) and genes in deregulated pathways (*GSVA*)

**Table 2:** Accuracy of RF and SVM classifiers on the neuronal dataset

Genes selected	Accuracy (%)^a^		MCC^^^	
	SVM	RF	SVM	RF
All genes^b^	95.3	76.9	0.91	0.00
GSVA feature enrichment	98.5	76.9	0.87	0.00
sRAP	100	76.9	1.00	0.00
SVM-RFE	100	100	1.00	1.00
RF-based Positive MDA	100	76.9	1.00	0.00
*T*-test	100	97.0	1.00	0.91

The accuracy of the SVM predictors were obtained from LOO cross validation. SVM and RF classifiers were constructed with each set of data listed in Table 2

^a^All percentages are rounded off to three significant figures

^b^Transcripts with a total expression of zero and/or having more than six samples with expression levels less than one were excluded

^^^Matthews correlation coefficient (MCC) rounded to 2 decimal places

### SVM and RF-based classification of neuronal cell types with enhanced feature selection

In this study, a total of five different feature selection techniques were employed in for dimensionality reduction and they are pathway-based selection by GSVA, statistical-based selection by sRAP and *T*-test approaches and ML-based selection by SVM-RFE and RF-based positive MDA approaches. The number of transcripts selected by each feature elimination method can be found in Table 1. Additionally, the corresponding accuracies and MCC of each classifier are listed in Table 2.

The feature selection process decreased the number of transcripts analysed by ~60% to more than 95%. The best classifier constructed was that with features selected by SVM-RFE. This selection gave the best prediction accuracies and MCC, 100% and 1 respectively, for both the SVM and RF classifiers. GSEA has enabled scientists to identify sets or group of deregulated genes where preliminary insights to alternation of cellular mechanisms under different biological conditions can be studied [10]. Given the usefulness of such a methodology, in this paper, we explore the impact on GSEA gene selection and prediction accuracy. Classifiers built with on GSVA-enriched genes did not considerably increase the prediction accuracy of the classifiers as a mere 3.2% increase in accuracy of the SVM predictor was obtained.

Nevertheless, it is interesting to note that RF classifiers generally have a lowered level of accuracy and a poor MCC value as compared to the cognate SVM models. For example, the RF classifier constructed using RF-based Positive MDA gene selection approach have an accuracy of 76.9, ~23% lower than that of the SVM classifier built with the same data. Also, the SVM classifier produces a perfect predictor (MCC = 1) while that of the RF classifier performs no better than random prediction (MCC = 0). This observation could be an indication of the short-coming of tree-based ML methods to build high quality classifiers with single-cell RNA-seq expression data.

### Network-level differences between NPCs and neuronal cells and their biological relevance in neuronal development

Gene regulatory networks (GRNs) among these transcripts inferred from RNA-seq expression profiles of SVM-RFE genes are useful in the investigation of system-level differences between the two cell types. Hub-genes (genes/transcripts that have a large number of regulatory interactions with other genes/transcripts) identified in GRNs might play potentially key roles in the maintenance of a particular cellular state. Thus, “differential hub genes (DHGs)” that are hub-genes/transcripts in neuronal cells (or NPCs) but not hub-genes in NPCs (or neuronal cells) could have important roles to differentiate the two cell types.

**Fig. 3:**
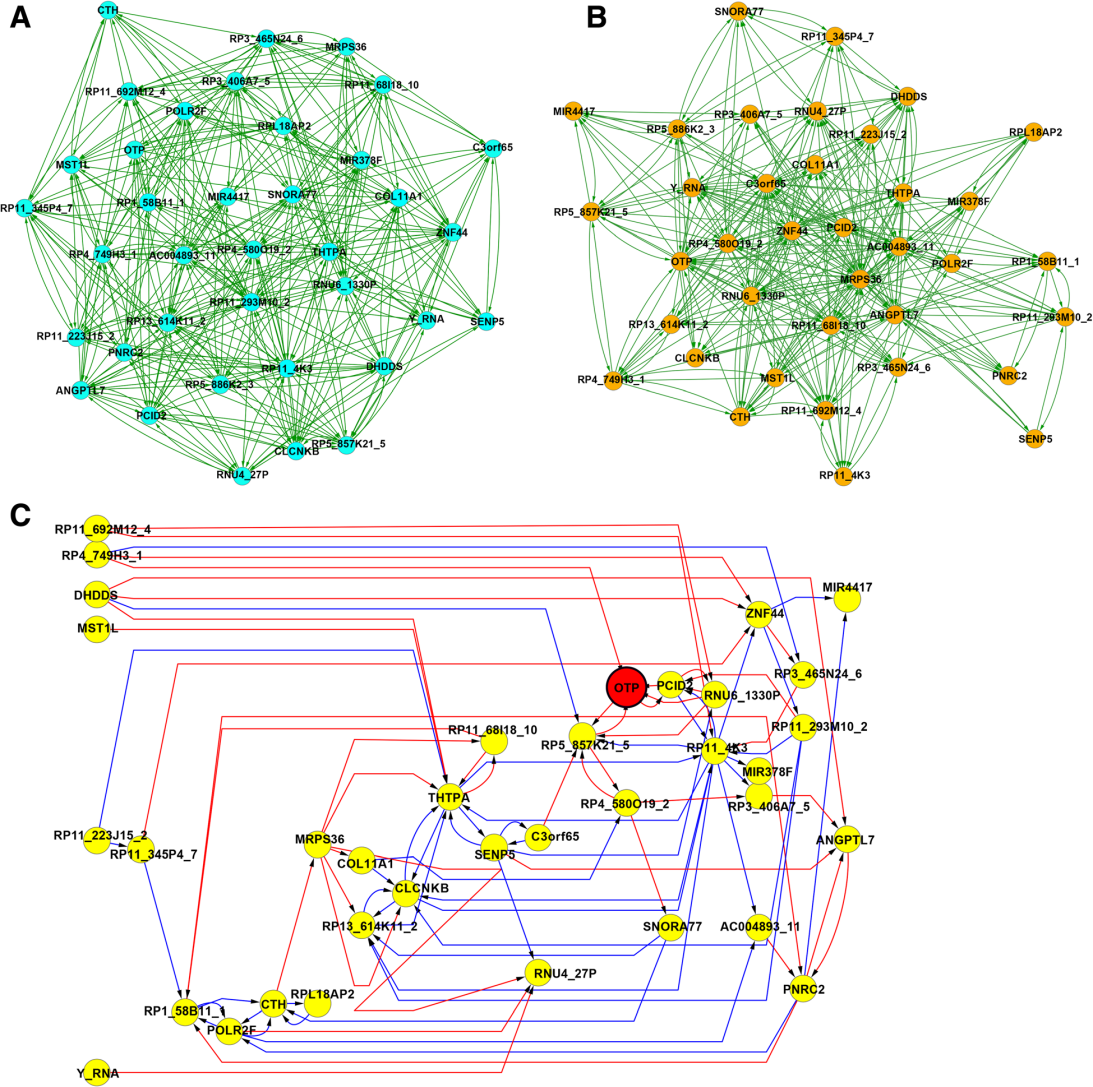
GRN in NPCs (**a**) neuronal cells (**b**) and differential GRN between the two cell types. **a** GRN in NPCs. Nodes represent transcripts, while links between two nodes represent regulatory interactions between two transcripts in NPCs. Gene regulatory interactions with high confidence score (confidence score > 0.75) in NPCs are shown in the diagram. **b** GRN in neuronal cells. Nodes represent transcripts, while links between two nodes represent regulatory interactions between two transcripts in neuronal cells. Gene regulatory interactions with high confidence score (confidence score > 0.75) in neuronal cells are shown in the diagram. **c** Differential GRN between two cell lines. Nodes are transcripts. Red links represent gene regulatory interactions that are activated in neuronal cells but not activated in NPCs, while blue links represents those activated in NPCs but not in neuronal cells. In this diagram, we assumed that a regulatory interaction is activated in neuronal cells (or NPCs) but is not activated in NPCs (or neuronal cells), if difference in confidence score between the two cell types is greater than 0.75, eg, an interaction whose confidence scores are 0.99 and 0.20 in neuronal cells and NPCs, respectively. Note that OTP is a representative DHG (see Table 3) and thus the gene is highlighted in red

In order to investigate network-level difference (eg, DHGs) between the two cell types, GRNs were inferred (see Methods for more details) in neuronal cells (see Fig. 3a), NPCs (see Fig. 3b) from RNA-seq expression profiles and the structure of the two GRNs were subsequently compared (Fig. 3c). As observed, a large number of regulatory interactions are activated in one cell type but are not activated in the other cell type (blue links represents regulatory interactions activated in NPCs but not in neuronal cells, while red links represents those activated in neuronal cells but not in NPCs). For example, several interactions of the *Homeobox protein orthopedia* (OTP) gene (red-colored node in Fig. 3c) are activated in neuronal cells but not in NPCs and this is indicative that OTP gene is a potentially important gene which is possibly regulated in neuronal cells but not in NPCs.

In order to identify DHGs between the two cell types, we used a representative network metric, “degree”, which is defined as the number of links to the transcript. For weighted network, *d*_*i*_, degree of a gene *i* is defined as, *d*_*j*_ = ∑_*j* = 1_^*N*^ *W*_*i*,*j*_, where *N* is number of transcripts in a GRN and *w*_*i,j*_ is weight (in this study, we used confidence score for a link as weight) for a regulatory interaction between two genes *i* and *j*. If a transcript have high-degree in a cell type, such transcript is defined as hub genes/transcripts in the cell type. In order to identify DHGs, for each of genes, we calculated difference in degrees between two cell types. For example, degree of MRPS36 in neuronal cells and that in NPCs are 57.08 and 33.99 respectively, and the degree difference of MPS is 23.09 (= |57.08 – 33.99|). Then, we ranked the transcripts according to their degree difference (Table 3). In Table 3, highly ranked transcripts (transcripts with high degree difference) are DHGs and may play an important role in differentiating neuronal cells from NPCs.

**Table 3:** Degree of difference between neuronal cells and NPCs in different genes

Genes	Degree in neuronal cells^a^	Degree in NPCs^a^	Degree difference between neuronal cells and NPCs^a^
MRPS36	57.1	34.0	23.1
RP11_4K3	23.0	44.0	21.0
SENP5	19.9	35.7	15.8
CLCNKB	23.9	39.6	15.7
POLR2F	23.7	39.2	15.4
OTP	48.3	33.1	15.2
RP1_58B11_1	24.6	39.3	14.7
RP11_293M10_2	32.4	45.9	13.5
RP3_465N24_6	25.6	39.1	13.5
SNORA77	30.0	43.2	13.2
C3orf65	50.8	38.0	12.8
ANGPTL7	49.7	37.9	11.8
RP4_580O19_2	47.2	36.9	10.2
RNU4_27P	45.1	35.0	10.0
COL11A1	25.8	35.3	9.46
RP11_68I18_10	47.9	39.3	8.67
Y_RNA	44.1	35.8	8.32
THTPA	29.9	37.5	7.61
ZNF44	45.7	38.6	7.11
CTH	28.9	35.9	7.02
RP11_692M12_4	42.8	35.8	7.02
RP11_345P4_7	32.0	38.8	6.82
RP13_614K11_2	36.5	43.3	6.81
MIR4417	30.7	37.2	6.55
RP11_223J15_2	36.7	42.5	5.77
RNU6_1330P	47.1	41.4	5.67
AC004893_11	45.1	39.9	5.23
RP3_406A7_5	33.4	37.5	4.06
MIR378F	39.1	35.5	3.63
PNRC2	33.7	30.6	3.15
RP5_886K2_3	43.5	40.6	2.89
RP5_857K21_5	34.9	33.2	1.61
PCID2	35.8	37.3	1.47
MST1L	38.5	37.4	1.00
RP4_749H3_1	39.5	38.9	0.624
RPL18AP2	36.0	36.6	0.573
DHDDS	41.1	40.7	0.414

^**a**^Degree of difference is corrected to three significant figures

Among the top ranked DHGs, we identified two potential key transcripts (the mitochondrial ribosomal protein S36 (MRPS36) and OTP) which could be responsible for the differentiation of NPCs from neuronal cells. MRPS36 is reported to be important in the maintenance of an undifferentiated state as overexpression of MRPS36 retards cell proliferation and delays cell cycle, helping cells maintain their undifferentiated state [11]. Similarly, the protein OTP is reportedly expressed in the hypothalamus during mammalian embryonic brain development and is a key determinant underlying FezI and Pac1-mediated of hypothalamic neural differentiation [12, 13]. These results indicate that the putative transcripts present within the top ranked DHGs could act as candidate targets for further experimental validation of their role in neuronal development. It is pertinent to note that as shown in Table 3, several of the SVM-RFE genes did not have significant difference in network property (degree) between the two cell types. This can signify that at the network level, all the SVM-RFE genes (DHGs) may not play biologically relevant role in differentiating the two cell types.

## Discussion

The advancement of high throughput sequencing technologies has brought about an unprecedented ability for scientists to analyze the highly complex eukaryotic transcriptome by RNA-Seq. As compared to its predecessors, RNA-Seq has a very high signal-to-noise ratio and very large dynamic range. Reproducibility of RNA-Seq sequencing is also high and is able to provide high correlation across biological and technical replicates [14–16]. Further, single-cell RNA-seq techniques were developed, allowing finer insights to be elucidated with respect to the dynamics of disparate cellular differentiation, responses to stimulation and the stochastic nature of transcription within individual cells within a tissue or a tumour. Though it is still expensive to carry out RNA-seq sequencing in the current paradigm, it is expected that the sequencing cost will significantly decrease within the next few years [17].

In view of the impending information overflow, there is a concurrent need to develop more efficient techniques for the analysis such big data, especially for the construction of predictive models which can aid the identification and classification of different cell types as described in this paper. This work is one of the first to analyse single-cell RNA-seq profiles for the construction of predictive classifiers for neuronal cells and NPC. Also, classification accuracy of different models, built with features selected by different methods (ML based, GSEA or traditional DE genes based methods), have also been critically assessed. Further, we integrated the classification results with a network inference pipeline to infer potential regulatory network amongst genes/transcripts (GRN) and identify network signatures for specific cell types.

Four key insights were obtained from this piece of work. First, transcripts selected by ML algorithm build better classifiers with enhanced accuracy, up to 100%, as compared to DE genes selected by traditional methods (sRAP and GSVA). Second, models built with differentially expressed transcripts selected from biological pathway-based methods (GSVA) proved to be inferior to that of models built with highly deregulated genes identified by traditional statistical means (sRAP and *T*-test). While pathway-based techniques are able to lend biological relevance to selected genes, genes selected by such methodology might not be able to capture gene expression signature of the disparate cell types studied. Thirdly, accuracy differs between classifiers built by different ML algorithms where RF, as compared to SVM, is unable to produce high accuracy for the high dimensional data analysed in this paper. Finally, the system-level analysis of the set optimal transcripts, using GRN inference analysis, is useful in the identification of hub transcripts which defines the different cell types investigated. Candidate transcripts identified by GRNs have potential biological correlates which are important in providing biological insights to cellular development. It is believed that such a workflow is extensible and applicable to other single-cell RNA-seq expression profiles like that of the study of the cancer progression within the highly heterogeneous cancer cells within a tumour [18].

## Conclusion

The advancement in sequencing technologies have always brought along immense computational challenges to accurately and rapidly analyse the large amount of data generated from such experiments. Single-cell sequencing will inevitably become the gold standard for the study of genetic/transcriptomic aberrations, thus, concurrent efforts need to be placed in parallel to devise computational pipelines which can effectively analyse such big data. In addition to the use of legacy algorithms passed down for the era of microarray data analysis, there is a need to inject novelty and creativity in the analysis of single-cell RNA-seq data. This can be achieved using the combination of machine learning algorithms like SVM and RF and network reconstruction algorithms as reported in this paper. We have demonstrated that predictors built from transcripts selected using machine-learning based feature selection techniques which outperforms the commonly used statistical techniques or geneset-based approaches. Also, the novel incorporation of network reconstruction techniques have led to the identification of existing interactions and also potentially new interaction networks are identified which can be further validated in a smaller number of wet lab experiments as candidate biomarker genes. We believe that such a pipeline is extensible to other single-cell RNA-seq datasets, including those of tumor samples where the intricate transcriptomic complexity of the highly heterogeneous tumor can be unravelled for the design of personalized treatment for individual patient.

## Methods

### Data preparation for Single-cell RNA-Seq

Single-cell RNA gene expression profiles of neural cells from Pollen et al. [19] were used for this study as training data for the SVM/RF classifiers and will be called the “Fluidigm neural dataset” in this study. The data contained expression profiles of four neuronal cell populations, 65 samples in total, including (i) neural non-progenitor cells (NPCs), (ii) cells from the germinal zone of human cortex at gestational week (GW) 16 (GW16), (iii) 21 (GW21) and (iv) a subset of cells at GW21 which were furthered cultured for 3 weeks (GW21 + 3). Raw reads, obtained from the NCBI Sequence Read Archive (http://www.ncbi.nlm.nih.gov/sra) [20] under accession number SRP041736 [3], were mapped to the reference genome using Tophat2 (v2.1.0) [21] and were subjected to Fragments per Kilobase of Exon per Million Fragments Mapped (FPKM) normalization, using cuffdiff (v2.2.1) [22], prior to downstream analysis. Expression levels were logged prior to filtering. Genes having log FPKM of greater than one in more than six cells were included in the analyses. Samples were assigned labels of NPCs and those of the neuronal cells (inclusive of expression data obtained from cells of GW16, GW21 and GW21 + 3).

### Construction of support vector machine (SVM) and random forest (RF) predictive models for the identification of fetal neocortical neuronal cells from NPCs

Classification accuracies of each disparate dataset were explored using two different machine learning algorithms - Support Vector Machine (SVM) and Random Forest (RF).

The SVM predictors were built with the LIBSVM package [23] and the RF predictors were built with the randomForest package in the R programming environment [24].

The detailed methodology for the construction of a SVM classifier can be obtained from the article by Burges [25] and a brief description of the SVM algorithm from Wee et al. [26]. Briefly, the SVM algorithm is based on the structural risk minimization principle from statistical learning theory [27]. A set of positive (single-cell RNA-Seq neocortical transcription data) and negative (single-cell RNA-Seq NPC transcription data) examples were represented by the feature vectors *x*_*i*_ (i = 1, 2,....N) with corresponding labels *y*_*i*_ ∈ {+1,-1}. To classify the data as NPCs or neuronal cells, the SVM trains a classifier by mapping the input samples, using a kernel function (radial basis function (RBF) in this study), onto a high- dimensional space, and then seeking a separating hyperplane that differentiates the two classes with maximal margin and minimal error. Parameter optimization was carried out for *g*, which determines the capacity of the RBF kernel, and the regularization parameter *C* using leave-one-out (LOO) cross-validation. The optimal *g* and *C* values obtained from the optimization processes were used subsequently for training the entire training set to create the final SVM classifier.

RF is a tree-based classifier where classification is carried out by aggregating the votes for all trees built from different subsamples, randomly selected, with replacement, within the training set, from the training dataset. As the classifier is built by aggregating a large number of different decision trees, predictors built with the random forests algorithm is expected to have low variance and low bias. The number of trees (T) was set to 20,000 and the number of features to consider at each split in the decision tree (m) obtained from the optimization processes were used subsequently for training the entire training set to create the final RF classifier [28, 29].

### Feature extraction and dimensionality reduction

Additionally, dimensionality reduction was carried out to obtain optimal subsets of gene/features for classifier construction and they are as listed below.

(i) Selection of genes from deregulated pathways using geneset enrichment analysis (GSEA). A non-parametric, unsupervised G was carried out with the Gene Set Variation Analysis (*GSVA*) package [30] in the R programming environment [24]. The original ensemble gene (ENSG) identifiers were mapped to their cognate HUGO Gene Nomenclature Committee (HGNC)/Uniprot identifiers using the biomaRt package in R [31]. This was carried out in order to permit the mapping of genes to that of the curated C2 geneset (September 2014), obtained from the Broad Institute’s Molecular Signatures database version 4.0 (MSigDB) [10], for gene set analysis. Manual curation of the HGNC/Uniprot identifiers was subsequently carried out to obtain a curated list of identifiers. Genes with ENSG codes that were not matched to any symbols were removed. Also, gene fragments sharing the same symbol were excluded from analysis. The identities of the up and down-regulated pathways (*p*-value < 0.005), together with the corresponding genes within these genesets, were identified and reported.
(ii) Selection of differentially expressed (DE) genes using the R package Simplified RNA-Seq Analysis Pipeline (*sRAP*) [32, 33].
(iii) Selection of a subset of genes with the highest ranking criterion based on SVM-based classification [34] (SVM-RFE) using the R package *pathClass* [35]. SVM-RFE is an iterative gene selection process where features, expression values of different genes obtained from single-cell RNAseq experiments, with the smallest ranking criterion are recursively removed when the ranking criterion for all features are computed from the SVM-classifiers.
(iv) Selection of genes with positive mean decrease in accuracy (MDA) from RF analyses where selected feature genes are deemed to reduce classification error.
(v) Selection of DE genes using two-tailed *T*-test based analysis using R package *limma* [36] (*p*-value < 0.05).

### Evaluation of model performance

A set of statistical variables were established to evaluate the performance, Accuracy and Matthews correlation coefficient (MCC) [37], of the SVM and RF classifiers. Only LOO cross validation was carried out for the SVM classifiers.

### Inference of gene regulatory networks in neuronal cells and NPCs

A plethora of network-inference algorithms are now available and have been used to infer GRNs from gene expression datasets. As mentioned in Marbach et al. [38] and Hase et al. [39], different network-inference algorithms have different strength and weakness and complement with each other. Thus, by integrating heterogeneous network-inference algorithms, we can take advantage of their strengths to recover high-quality gene regulatory networks [38, 39].

Therefore, in this study, we selected 14 representative network-inference algorithms that are based on heterogeneous statistical techniques and integrated results from the selected algorithms. The selected algorithms includes six mutual information based methods (ARACNE [40], CLR [41], MRNET [42], RELNET [43], C3NET [44], and BC3NET [45]), two correlation based method (Spearman’s correlation and Pearson’s correlation [43]), one Bayesian network based method (SiGN-BN [46]), two random forest based method (GENIE3 [47] with two different parameter settings, see Table 4 for the details), two regression based method (TIGRESS [48] with two different parameter settings, see Table 4 for the details), and one method with both of ordinary differential equation based recursive optimization and mutual information (NARROMI [49]). The set of 14 algorithms includes several high-performance algorithms, ie, GENIE3 is the winner of both of DREAM4 (DREAM, Dialogue on Reverse Engineering Assessment and Methods) and DREAM5 network inference challenges [38, 47], while, in DREAM5, TIGRESS and CLR (and MRNET) are the best performer among regression techniques and that among mutual information techniques, respectively [38]. Zhang et al. demonstrated that NARROMI outperforms GENIE3 [49].

**Table 4:** Parameter used to optimize each network-inference algorithms

Network-inference algorithms	Parameter optimization setting^c^
GENIE3-A^a^	K = “all”, nb.trees = 10,000
GENIE3-B^a^	K = “sqrt”, nb.trees = 10,000
TIGRESS-A^b^	scoring = “area”
TIGRESS-B^b^	scoring = “max”
ARACNE	eps = 0.1
BC3NET	boot = 10, alpha1 = 0.99, alpha2 = 0.99
SiGN-BN	Number of iteration of bootstrap method = 1,000

^a^GENIE3-A and -B represent two different parameter settings for GENIE3 algorithm used in this study

^b^TIGRESS-A and -B represent two different parameter settings for TIGRESS algorithm used in this study

^c^We used default settings for parameters that are not shown in this table

### Integration of results from multiple network-inference algorithms

In this study, we have used a computational framework, “Top1net”, to integrate results from the selected 14 network-inference algorithms [39].

Each of individual network-inference algorithms calculates confidence score for each gene pair and, an interaction between a gene pair with higher confidence score is more likely to be true positive interaction [39, 40, 50]. Top1net applies bagging method introduced by Breiman [51], to integrate confidence scores for each of gene pairs from multiple individual network-inference algorithms [39]. Top1net assumes that, if at least one network-inference algorithm assigns high confidence score to a gene pair, one gene in the pair has a regulatory interaction with another gene [39]. As network-inference algorithms tend to assign high confidence scores to true positive interactions, Top1net would recover a large number of true positive interactions in a GRN. The procedure of Top1Net is composed of three steps.

#### Step 1

From an expression dataset, an individual network-inference algorithm assigns confidence score for each of gene pairs and the gene pairs are ranked according to their confidence scores, ie, a gene pair with highest confidence score has the rank value of 1.

#### Step 2

We normalized ranked scores from each algorithm by scaling from 0 to 1 and used the normalized ranked scores (NRSs) as confidence scores by the algorithm. If a pair of genes *i* and *j* has rank value of *g*_*i,j*_ by an algorithm, the *NRS*_*i,j*_ of the gene pair by the algorithm is defined as, $NR{S}_{i,j}=\frac{N\left(N-1\right)+1-{g}_{i,j}}{N\left(N-1\right)}$, where *N* represents the number of genes in the gene expression dataset.

#### Step 3

We integrate NRSs from the algorithms by Top1net. For example, if we used the 14 network-inference algorithms to calculate 14 NRSs for each gene pairs. For each gene pairs, Top1net used the highest NRS among 14 NRSs as the confidence score of the gene pairs. For example, if the algorithms assign 14 NRSs, 0.98, 0.85, 0.8, 0.69, 0.65, 0.63, 0.62, 0.61, 0.58, 0.55, 0.53, 0.51, 0.50 and 0.35 for the gene pair, Top1net used 0.98 as the confidence score for the interaction between the gene pair.

#### RNA-seq expression profiles for GRN inference

Only 37 genes identified by SVM-RFE method were used for the inference of gene regulatory interactions within neuronal cells and NPCs as a single gene, RP4_803A2_1, was excluded for having expression values of 0 across all NPC samples.

#### Packages and parameters for individual network inference algorithms

To infer GRNs by individual algorithms, we used MINET package [52] for ARACNE, CLR, MRNET and RELNET, c3net packages for C3NET, bc3net packages for BC3NET, source code obtained from http://www.montefiore.ulg.ac.be/~huynh-thu/software.html for GENIE3, source code obtained from GP-DREAM network inference website (http://dream.broadinstitute.org/) for TIGRESS, and source code from http://comp-sysbio.org/narromi.htm for NARROMI. For SiGN-BN [28], we used software on the super-computing resource that was provided by Human Genome Center, the Institute of Medical Science, and the University of Tokyo. For PCC and SCC, we used R function (“cor” function) to calculate Pearson’s and Spearman’s correlation coefficient.

Nine, out of the 14, algorithms required optimization and the parameter settings for the nine network-inference algorithms are shown in Table 1. More information on algorithm customization can be found in references [40, 45–49] and also within the manuals written for the individual algorithms.
